# A genotypic HIV-1 proviral DNA coreceptor tropism assay: characterization in viremic subjects

**DOI:** 10.1186/1742-6405-11-14

**Published:** 2014-05-21

**Authors:** Jennifer Brown, Harold Burger, Barbara Weiser, Richard B Pollard, Xiao-Dong Li, Lynell J Clancy, Russell E Baumann, Amy A Rogers, Hasnah B Hamdan, Rick L Pesano, Ron M Kagan

**Affiliations:** 1 Division of Infectious Diseases, Department of Internal Medicine, University of California, Davis Medical Center, 4150 V. Street, PSSB-G500, Sacramento, CA, USA; 2 University of California, Davis, School of Medicine, Davis, CA, USA; 3 Department of Infectious Diseases, Quest Diagnostics Nichols Institute, San Juan Capistrano, CA, USA

**Keywords:** HIV-1 diagnostic tests, HIV-1 tropism, HIV-1 proviral tropism

## Abstract

**Background:**

HIV-1 coreceptor tropism testing is used to evaluate eligibility for CCR5 antagonist therapy. However, HIV-1 RNA-based tests are not suitable for virologically suppressed patients, therefore the use of proviral DNA tropism testing has been investigated. We describe a novel proviral DNA-based genotypic tropism assay and compare its performance to that of a sensitive HIV-1 RNA-based genotypic test.

**Methods:**

Tropism was determined using HIV-1 plasma RNA and proviral DNA from 42 paired samples from patients with plasma viral loads ≥1000 HIV-1 RNA copies/mL. Proviral DNA sample types included whole blood, separated peripheral blood mononuclear cells resuspended in phosphate-buffered saline and peripheral blood mononuclear cells resuspended in spun plasma. The HIV-1 envelope V3 region was PCR-amplified, sequenced in triplicate, and analyzed for tropism with the geno2pheno algorithm using a 10% false-positive rate (FPR).

**Results:**

Amplicons were obtained from proviral DNA and plasma RNA in 41/42 samples. Tropism predictions were highly concordant (93%–98%) between proviral DNA and plasma RNA, regardless of the proviral DNA isolation method. Non-R5 proviral DNA results were obtained for 100% of patients with detectable non-R5 plasma HIV-1 RNA results. Geno2pheno FPRs for proviral DNA and plasma RNA were highly correlated (Spearman rho = 0.86).

**Conclusions:**

Our findings demonstrate that proviral DNA tropism determinations from whole blood or peripheral blood mononuclear cells were highly concordant with plasma HIV-1 RNA tropism determinations. This assay may be useful for screening virologically suppressed patients for CCR5-antagonist eligibility and for research purposes.

## Introduction

Human immunodeficiency virus type 1 (HIV-1) requires contact with two receptors to gain entry to host cells and initiate infection: CD4 is the primary receptor and the chemokine receptors CCR5 and CXCR4 serve as secondary or co-receptors [1]. HIV-1 tropism refers to the co-receptors utilized by the virus and can be classified as R5, or X4 [1,2]. The major viral determinant of HIV-1 tropism is the V3 region of the envelope protein gp120, the portion of the viral envelope that interacts with the CCR5 and CXCR4 receptors [1-6]. The accurate determination of tropism is clinically important because CCR5 antagonists such as maraviroc are recommended only for HIV-1-infected individuals harboring exclusively CCR5-tropic virus.

Most tropism tests utilize plasma HIV-1 RNA to determine tropism in viremic, HIV-1 infected patients. Genotypic and phenotypic tropism assays of plasma HIV-1 are available as clinical tests and are recommended in US and European guidelines [7-9]. However, there is now increased emphasis on using HIV-1 proviral DNA (pvDNA) to ascertain tropism. Plasma viral RNA represents currently replicating HIV-1, while pvDNA is intracellular viral DNA that represents a population of archived virus [7-10]. pvDNA can be extracted from the blood of HIV-1 patients with undetectable RNA viral loads in plasma [10,11]. This permits tropism testing to extend to virologically suppressed patients on effective antiretroviral regimens, who may be candidates for CCR5 antagonist therapy because of the need for regimen switches or simplification.

Here we evaluated a genotypic test for proviral tropism determination based on sequencing HIV-1 pvDNA encoding the V3 region. HIV-1 proviral tropism was first determined in blood samples obtained from a diverse, well-characterized group of viremic patients followed at the Center for AIDS Research, Education and Services (CARES) in Sacramento, CA. We then evaluated concordance of tropism predictions using pvDNA and plasma viral RNA obtained from paired specimens.

## Results

HIV-1 V3 loop amplicons were successfully obtained from both pvDNA and plasma RNA in 41 of the 42 HIV-1–positive samples (98%). No HIV-1–negative control samples yielded amplification products. The median subject age was 40 years old (IQR: 31–46), and most (40/42) subjects were male. Subjects had a median plasma HIV-1 viral load of 4.3 (IQR: 3.3, 5.0) log_10_ copies/mL and a median of 352 (IQR: 192, 617) CD4+ T-cells/μL.

**Figure 1:**
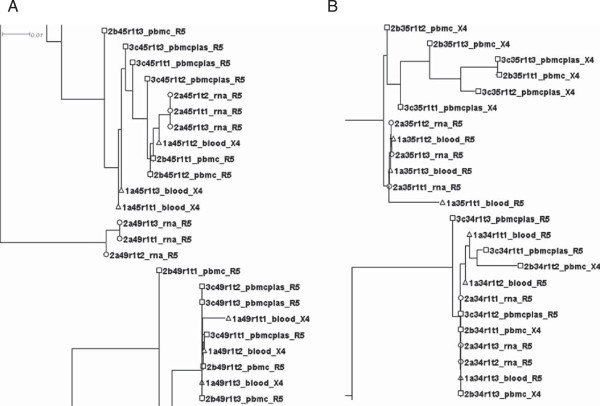
**Phylogenetic analysis of four samples with discordant tropism results. A**: samples 49 and 45. **B**: samples 34 and 35. Circles: V3 sequences from RNA; triangles: pvDNA V3 sequences from whole blood; squares: pvDNA V3 sequences from PBMCs.

Of the 41 patients with successfully amplified HIV-1 RNA and pvDNA, 14 (34%) had non-R5 results (X4 –tropic virus detected) on RNA testing (Table 1). Relative to the plasma RNA test, the pvDNA test was 100% sensitive for non-CCR5 virus (14/14) regardless of the sample types used for DNA extraction. However, pvDNA testing resulted in additional non-CCR5 predictions using whole blood (n = 3), PBMCs (n = 2), and PBMCs resuspended in plasma (n = 1). Phylogenetic tree analysis of the five samples that had at least one discordant result between sample types showed no evidence of inter-sample contamination (Figure 1, Additional file 1: Figure S1 and Additional file 2: Figure S2). The replicate RNA and pvDNA sequences for each respective patient were clustered together. For sample 49 (RNA: R5, pvDNA whole blood: X4) the three RNA-derived V3 sequences were found in a cluster that was divergent from the pvDNA sequences, indicative of a distinct viral quasispecies in the plasma compartment (Figure 1A). Likewise, the plasma RNA sequences for sample 26 (concordant R5) also diverged from the pvDNA sequences for this patient (Additional file 1: Figure S1). The V3 sequences for sample 18 (concordant X4) were also divided between two divergent clusters (Additional file 1: Figure S1). For sample 35, the PBMC-derived (X4) V3 sequences branched separately from the whole blood (R5) and plasma RNA (R5) V3 sequences (Figure 1B).

**Table 1:** Tropism prediction concordance between 3 proviral DNA (pvDNA) sample types and plasma RNA in paired samples from 41 HIV-1 positive patients

**pvDNA source**	**X4, No. (%)**		**Concordance**	**Sensitivity**^**a**^	**Specificity**^**b**^	**Kappa**^**c**^
	**Test**	**Ref**^**d**^				
Whole blood (Tube 1a)	17 (41)	14 (34)	93%	100%	89%	0.85
PBMCs (Tube 2b)	16 (39)	14 (34)	95%	100%	93%	0.90
PBMCs + Plasma (Tube 3)	15 (37)	14 (34)	98%	100%	96%	0.95

^a^Sensitivity is defined as concordant X4 results between the test and reference methods/reference method X4s.

^b^Specificity is defined as concordant R5 results between the test and reference methods/reference method R5s.

^c^Kappa: a measure of inter-rater agreement: 0.41-0.60: moderate agreement; 0.61-0.80: good agreement; 0.81 – 1.0 excellent agreement.

^d^Plasma RNA testing was used as the reference method for all proviral DNA tests.

Thus, pvDNA testing yielded a higher proportion of non-CCR5 predictions than did RNA testing (Table 1). pvDNA testing showed exclusively CCR5 tropism in plasma and whole blood from 24 (56%) patients. Tropism predictions were highly concordant (93% to 98%) between pvDNA and plasma RNA, regardless of the sample type used for pvDNA isolation (Table 1).

The average geno2pheno FPRs for the replicate pvDNA and plasma RNA tropism determinations were strongly correlated. The Spearman rho correlation coefficient was 0.94 (95% CI: 0.90 – 0.97) for whole blood vs plasma RNA. There was a higher degree of variability between the FPRs for pvDNA isolated from PBMCs and from PBMCs resuspended in plasma compared to plasma RNA, with Spearman correlation coefficients of 0.86 in both cases (95% CIs: 0.76 – 0.92 and 0.75 – 0.92). The FPRs for the two PBMC sample types were highly correlated (Spearman rho = 0.96; 95% CI: 0.93 – 0.98).

Patients harboring non-CCR5 virus tend to have lower CD4+ cell counts than patients harboring exclusively CCR5 virus [12,13]. Although these differences were not statistically significant (Mann–Whitney test, p = 0.16) in our study owing to the small sample size, median (IQR) CD4+ counts were lower among non-CCR5 subjects (median, 316; IQR, 77–418 cells/μL) than in CCR5 subjects (median, 384; IQR, 212–687 cells/μL).

## Discussion

We report a novel genotypic pvDNA tropism assay that is based on population sequencing of the V3 region of HIV-1 pvDNA. Using paired, freshly drawn samples, we found high concordance in tropism predictions made using pvDNA and plasma RNA. This finding is consistent with the results of other studies, despite differing methods for determining tropism via HIV-1 envelope V3 region sequencing [10,14,15]. In addition, recent studies have also demonstrated a high degree of concordance between RNA and pvDNA tropism determinations in HIV-1 subtype C and HIV-2 [16,17]. Taken together, these findings support the use of pvDNA tropism testing as an alternative to RNA tropism testing.

Notably, we observed a high degree of concordance regardless of whether the pvDNA was extracted from whole blood or from PBMCs (either concentrated or resuspended in PBS). This is significant because, while whole blood may be the most convenient specimen type for tropism testing in the clinical setting, frozen PBMCs may be more favorable for research studies on archived specimens. Previous studies have shown that CXCR4-tropic virus is detected slightly more often with pvDNA than with plasma RNA [12,15,18]. This is because pvDNA comprises a heterogeneous population of archived genomes, whereas plasma RNA represents currently replicating HIV-1. Among the samples evaluated in this study, CXCR4-predicted sequences also were detected more often in pvDNA than in plasma RNA. Although most V3 sequences were well-conserved between the plasma and the PBMC compartments, some samples showed a much higher degree of diversity, with distinct subpopulations appearing in divergent clusters. A previous study that used ultradeep sequencing analysis of the V3 loop found that concordance between plasma and PBMC tropism was CD4 + −dependent, with 100% concordance when the count was above 350 cells/μL but only 74% when the count was below 50 cells/μL [19]. In the current study, the median CD4+ count was 352 cells/μL, consistent with the high level of observed concordance. One of three discordant samples (pvDNA non-R5, plasma RNA R5) had a CD4+ count below 350 cells/uL (195 cells/uL).

Current guidelines from Europe endorse the use of V3 loop sequencing of pvDNA to determine tropism, particularly for patients with low-level viremia or with viral suppression [9]. From a clinical standpoint, the ability to determine tropism from pvDNA allows testing to be extended to persons who are on effective antiretroviral therapy but who may be candidates for CCR5 antagonist therapy. This includes patients who are experiencing adverse effects from their current regimen and patients receiving complex regimens who may benefit from regimen simplification. In a pilot study of virologically suppressed patients who were switched to a maraviroc-containing regimen after a R5 pvDNA genotypic tropism test result, 82% of patients remained suppressed after 12 months [18].

In conclusion, we have characterized a HIV-1 pvDNA genotypic tropism assay using whole blood and PBMCs from HIV-1 infected subjects. Although this was a small study, a high degree of concordance was demonstrated between pvDNA and plasma viral RNA tropism determinations. This genotypic pvDNA tropism test may be useful as a surrogate for plasma RNA tropism testing to screen patients with suppressed or low level viremia for CCR5 antagonist eligibility. Additional clinical studies will be useful to further validate this approach. Furthermore, this assay may have value as a research tool for the examination of proviral HIV DNA in PBMCs.

## Methods

### Subject population

CARES is located in Sacramento, CA, and is affiliated with the University of California, Davis, Medical Center. It serves approximately 2,400, ethnically diverse, HIV-infected patients in an outpatient setting. Fifty-percent of the patients are non-Caucasian; 24% are Black/African-American, 18.6% Hispanic/Latino, 2.9% American Indian/Alaska Native, and 3.3% Asian/Native Hawaiian/Pacific Islander. Eighty-one percent are men, 18% are women and 1% are transgender. Approximately half of the patients (54%) are between the ages of 30–50 years old. The reported routes for HIV infection among the CARES population include: 58% men who have sex with men (MSM), 22% heterosexual, 15% intravenous drug use, and 1.0% hemophilia/blood transfusion/vertical transmission.

For this study, eligible subjects were HIV-infected patients >18 years old drawn from the CARES population with viral loads above 1000 HIV-1 RNA copies/mL. Subjects could be antiretroviral treatment-naïve or treatment-experienced. Forty-two HIV-positive subjects volunteered for the study. In addition, 7 healthy HIV-negative volunteers were chosen to provide negative control samples. The study was approved by the Institutional Review Board at the University of California, Davis Medical Center and all subjects gave written informed consent. All subjects received a US$5 gift card.

### Study design

**Figure 2:**
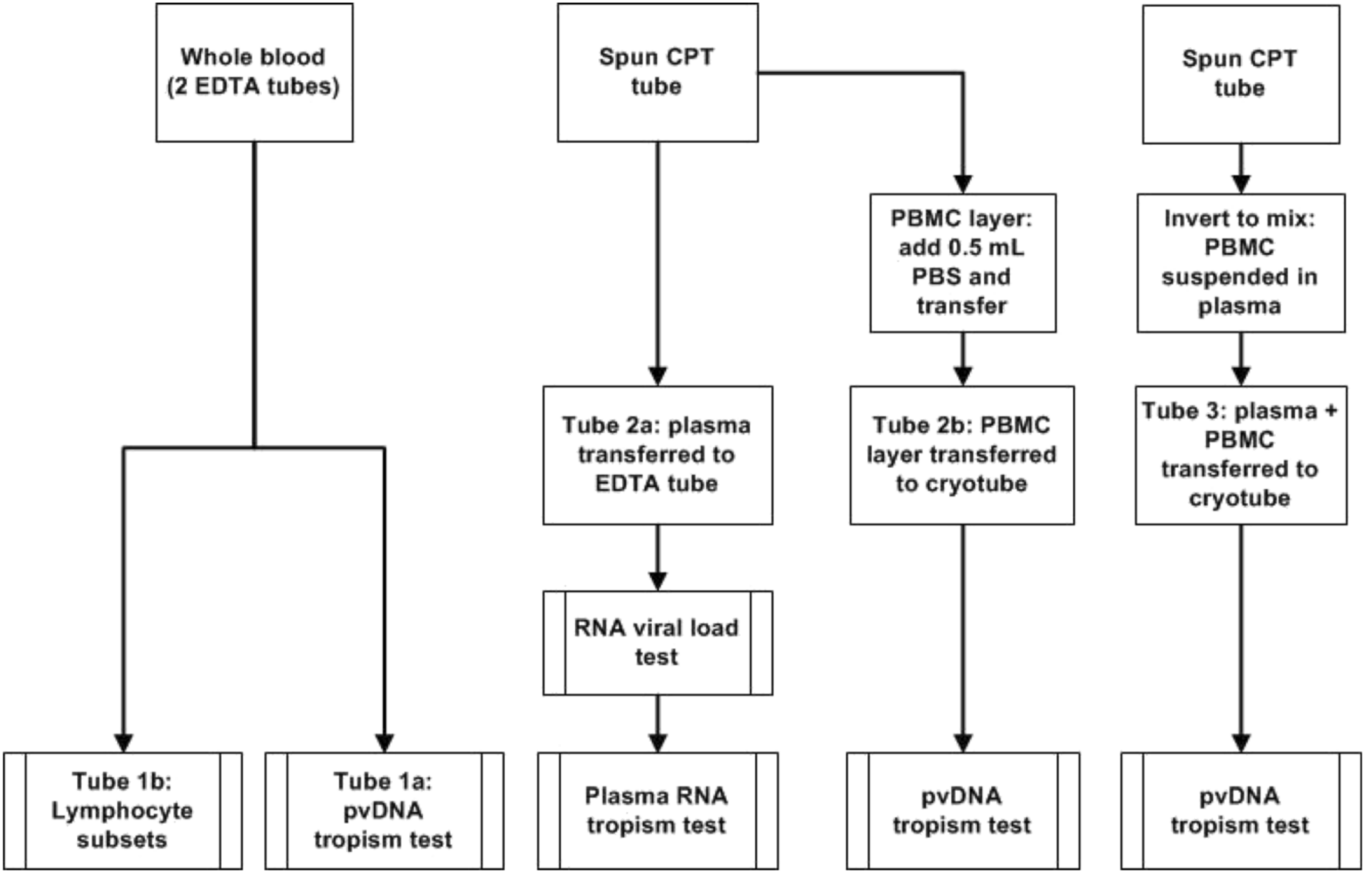
**Laboratory analysis of study samples.** Samples were collected and prepared for tropism analysis as described in Methods.

The study was designed to evaluate a novel HIV-1 pvDNA tropism assay developed in a clinical laboratory (Quest Diagnostics Nichols Institute, San Juan Capistrano, CA, USA) by assessing the concordance between pvDNA- and plasma RNA-based tropism determinations in viremic patients. Three sample types for pvDNA extraction were assessed: whole blood, separated PBMCs resuspended in phosphate-buffered saline (PBS), and peripheral blood mononuclear cells (PBMCs) resuspended in spun plasma (Figure 2). Plasma viral load and total, CD4 lymphocyte counts were also determined to gauge possible effects on the assay success rate or on concordance between sample types.

### Sample collection and preparation

Samples were collected between November 2011 and August 2012 at the CARES clinic from 42 HIV-positive and 7 healthy HIV-negative volunteers. Whole blood for pvDNA extraction and for lymphocyte count determinations was drawn into two 4 mL EDTA Vacutainer tubes (Becton Dickenson, Franklin Lakes, NJ, USA) and shipped at room temperature (Figure 2: Tubes 1a and 1b). Whole blood was also drawn into two 8 mL CPT Vacutainer tubes (Becton Dickenson). The tubes were centrifuged at 800 *g* for 30 minutes. Up to 3 mL of plasma for viral load determination and RNA tropism testing were removed from the first tube, transferred to an EDTA tube, and stored frozen at -70C (Figure 2: Tube 2a). To obtain PBMCs for pvDNA tropism testing, extra plasma was removed without disturbing the cell layer above the gel barrier; 0.5 mL PBS was then added to the CPT tube to resuspend the cell layer, which was transferred to a cryovial and stored frozen at -70C (Figure 2: Tube 2b). A further PBMC sample was obtained from the second spun CPT Vacutainer tube. The upper layer, containing the separated plasma and cell layer, was gently mixed and then transferred into a 15 mL conical tube and stored frozen at -70C (Figure 2: Tube 3).

### Plasma viral load measurements and lymphocyte counts

HIV-1 plasma RNA viral loads were determined using the Roche COBAS AmpliPrep/COBAS TaqMan HIV-1 test, version 2.0 (Roche Diagnostics Corp., Indianapolis, IN, USA). CD4+ and CD8+ T-cell percentages and absolute counts were determined by flow cytometry and standard hematological methods. These assays were performed at Quest Diagnostics Nichols Institute, San Juan Capistrano, CA, USA.

### Plasma RNA tropism determination

Tropism was determined for 3 independent replicates of viral RNA essentially as described elsewhere [2]. In brief, viral RNA extraction was performed, followed by reverse transcription and first-round PCR with forward and reverse primers SQV3F1 (HXB2 genomic coordinates 6855–6878) and CO602 (HXB2 genomic coordinates 7786–7817) in 3 independent replicates. Second-round PCR was performed with primers 2 F (HXB2 genomic coordinates 7062–7084) and 2R (HXB2 genomic coordinates 7350–7373). Population sequencing was performed with the second round PCR primers. Sequences were assembled with ReCALL software (BC Centre for Excellence in HIV/AIDS, Vancouver, BC) [20] and the 105 nt V3 loop region (codons 1–35) was utilized for tropism assignment using the geno2pheno algorithm with a false positive rate (FPR) of 10% (X4: FPR ≤10%) [21].

### Proviral DNA tropism determination

Total DNA was extracted from 0.5 mL whole blood or 0.3 mL PBMCs resuspended in PBS or the patients’ plasma on a Roche MagNA Pure system using the Large Volume MagNA Pure LC DNA Isolation Kit (Roche Diagnostics Corp.). Three independent replicates were amplified in 2 rounds of PCR and analyzed by population sequencing using the same priming strategy that was used for HIV-1 RNA tropism determination. Tropism assignment was performed with the geno2pheno algorithm at a 10% FPR as described for RNA tropism determination. A negative control sample (HIV-negative human plasma) was included in all runs. Positive controls for tropism status consisted of an R5 and an X4 first-round PCR amplicon prepared from pvDNA isolated from commercially available cultured R5 and X4 HIV-1 strains. Within assay and between assay reproducibility was 100% for tropism classification and the limit of detection (LOD_95_) for amplification of the pvDNA V3 loop was below 500 DNA copies/mL (Table 2).

**Table 2:** Proviral DNA tropism assay performance characteristics

	**Within assay precision**^**1**^		**Between assay precision**^**2**^		**LOD**_**95**_^**5**^	
Specimen type	Whole blood	PBMC	Whole blood	PBMC	Whole blood	PBMC
Tropism concordance^3^	100%	100%	100%	100%	N/A	N/A
DNA sequence concordance^4^	98.9%	98.9%	99.0%	98.6%	N/A	N/A
DNA copies/mL	N/A	N/A	N/A	N/A	480	360

^1^Three replicates of 3 clinical samples were tested in a single run. Two samples were classified as R5 and one was classified as X4.

^2^Three replicates of 3 clinical samples were tested on 3 different days in 3 runs. Two samples were classified as R5 and one was classified as X4.

^3^The tropism classifications for all replicates were 100% concordant with the original R5 or X4 classification.

^4^Nucleotide sequences for the replicates were compared to the consensus sequence and differences were tabulated. Mixed base calls (K, M, R, S, W, Y) accounted for the differences.

^5^The sensitivity standards were prepared by diluting purified, quantitated 963 bp HIV-1 proviral DNA amplicons in HIV-1 negative human whole blood or plasma and the LOD_95_ values were determined by probit analysis.

### Phylogenetic analysis

To rule out inter-sample contamination, all V3 sequences from viral RNA and pvDNA sample types, as well as the assay R5 and X4 controls were aligned using ClustalX v2.0 [22] and a neighbor-joining phylogenetic tree was created and visualized with DendroScope 3 [23].

## Abbreviations

CARES: Center for AIDS Research, Education and Services; FPR: False-positive rate; HIV-1: Human immunodeficiency virus type 1; MSM: Men who have sex with men; PBMCs: Peripheral blood mononuclear cells; PBS: Phosphate-buffered saline; pvDNA: Proviral DNA.

## Competing interests

Authors HB and BW are the inventors of six patented technologies for the determination of HIV tropism in clinical management. The patents are owned by Health Research Inc., the research foundation for the New York State Department of Health, and are licensed to Quest Diagnostics. Authors REB, RMK, AAR, HBH, and RLP are employed by Quest Diagnostics, a diagnostic testing company that offers diagnostic tests for HIV. Authors JB, LJC, XDL, and RBP have no conflicts of interest.

## Authors’ contributions

Conception and design: HB, REB, RMK, XDL, RBP, RLP, AAR, BW. Acquisition of data: JB, REB, LC, XDL, AAR. Analysis and interpretation of data: HB, JB, RMK, BW. Statistical analysis: RMK. Drafting the manuscript or revising it critically for important intellectual content: HB, JB, RMK, LC, XDL, RBP, BW. All authors read and approved the final manuscript.

## Supplementary Material

Additional file 1: Figure S1 Neighbor-joining phylogenetic tree for all replicate V3 loops sequenced in this study. Circles: V3 sequences from RNA; triangles: pvDNA V3 sequences from whole blood; squares: pvDNA V3 sequences from PBMCs. Click here for file

Additional file 2: Figure S2 Phylogenetic analysis of sample 43. Circles: V3 sequences from RNA; triangles: pvDNA V3 sequences from whole blood; squares: pvDNA V3 sequences from PBMCs. Click here for file
