# Supplementary figures and images for: A genotypic HIV-1 proviral DNA coreceptor tropism assay: characterization in viremic subjects
Source: AIDS Res Ther. 2014 May 21;11:14. doi: 10.1186/1742-6405-11-14 (PMC4045881; doi:10.1186/1742-6405-11-14)

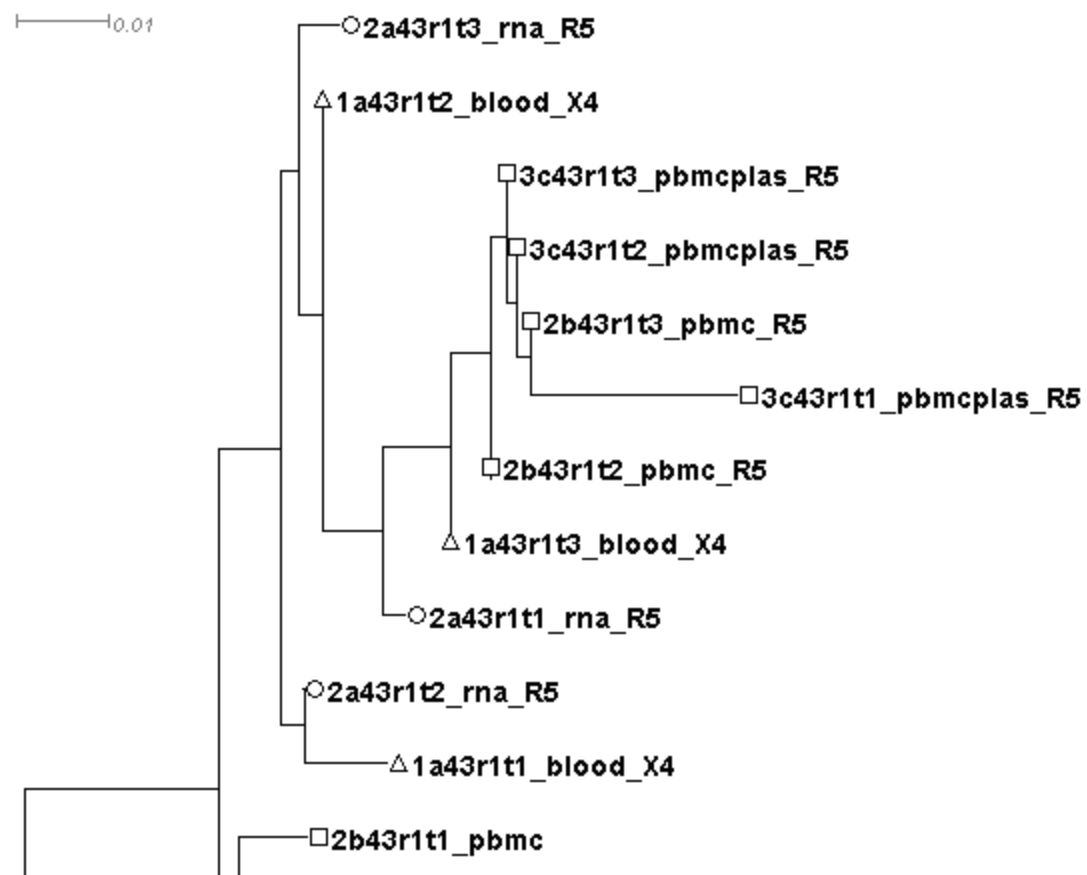

Supplement: Additional file 2: Figure S2 — Phylogenetic analysis of sample 43. Circles: V3 sequences from RNA; triangles: pvDNA V3 sequences from whole blood; squares: pvDNA V3 sequences from PBMCs. [file 1742-6405-11-14-S2.pdf]
